# Draft Genome Sequence of Stenotrophomonas maltophilia MDMC339, Isolated from Soil of Merzouga Desert in Morocco

**DOI:** 10.1128/MRA.00634-20

**Published:** 2020-08-06

**Authors:** M. W. Chemao-Elfihri, Amina Manni, Meriem Laamarti, Souad Kartti, Abdelmounim Essabbar, Tarek Alouane, Loubna Temsamani, Jamal-Eddine Eljamali, Mouna Ouadghiri, Nargisse El Hajjami, Laila Sbabou, Lahcen Belyamani, Azeddine Ibrahimi, Abdelkarim Filali-Maltouf

**Affiliations:** a Biotechnology Lab (MedBiotech), Bioinova Research Center, Rabat Medical & Pharmacy School, Mohammed V University, Rabat, Morocco; b Laboratory of Microbiology and Molecular Biology, Faculty of Sciences, Mohammed V University, Rabat, Morocco; c Emergency Department, Military Hospital Mohammed the Vth, Rabat, Morocco; d Bioinova Research Center, Rabat Medical & Pharmacy School, Mohammed V University, Rabat, Morocco; Georgia Institute of Technology

## Abstract

Here, we report the draft genome sequence of Stenotrophomonas maltophilia MDMC339, a strain able to survive in the difficult conditions imposed by the Merzouga desert. The analyzed genome contains 4,788,525 bp with 4,262 genes coding for proteins, including several genes related to stress.

## ANNOUNCEMENT

Merzouga is a small Saharan village in southeastern Morocco known for its arid climate and low rainfall. The region is characterized by a very hot climate with significant variations in temperatures between day and night. In such situations, living organisms have to cope with extreme temperatures, low humidity, and low nutrient availability. However, some bacteria implement several strategies to counter stress (1–3).

Stenotrophomonas maltophilia is a ubiquitous, Gram-negative, aerobic, rod-shaped, and mobile bacterium with some polar flagella (4, 5). It is also an opportunistic pathogen that has low sensitivity to many antibiotics (4, 6). Nevertheless, several strains have been described as producing plant growth-promoting substances or enzymes involved in bioremediation, thus bringing out its environmental benefit (7).

In this study, we report the draft genome sequence of Stenotrophomonas maltophilia MDMC339, isolated from nonvegetated dunes of the Merzouga region.

The sample was collected (at the coordinates –3.97852083325, 31.1093333325) in three replicates.

Bacterial isolation was done using 1 g of sand, which was suspended in 9 ml of physiological water and then shaken vigorously for 30 min. Serial dilutions (from 10^−3^ to 10^−7^ ml/ml) were made. Then, 100 μl of each dilution was spread on the surface of a petri dish containing nutrient agar medium supplemented with 50 μg/ml of amphotericin B. The dishes were then incubated at 28°C for 2 days. Once purified, the isolate was seeded on liquid nutrient broth and incubated overnight. Subsequently, the isolate was designated MDMC339 (Moroccan Desert Microorganisms Collection) and stored in 40% (vol/vol) glycerol solution at −80°C.

From the frozen stock, the culture process was carried out in 5 ml of nutrient broth at 28°C for 48 h. After centrifugation, the pellet was used for DNA extraction using the phenol-chloroform method as described by Chen and Kuo (8) and quantified using the Quantus fluorometer (Promega). Isolated bacterial genomic DNA was used both for bacterial identification using the 16S rRNA gene and for the whole-genome sequencing (WGS).

MDMC339 was identified as a Stenotrophomonas maltophilia isolate after 16S rRNA gene amplification using the universal primers FD1 (5′-CCGAATTCGTCGACAACAGAGTTTGATCCTGGCTCAG-3′) and RS16 (5′-TACGGCTACCTTGTTACGACTT-3′) followed by Sanger sequencing of amplicons (Genoscreen, Lille, France).

Using BLAST (9), the obtained 16S rRNA gene sequences showed more than 99% similarity to the 16S rRNA gene sequences of other S. maltophilia strains. More information about the identity of the bacterium was revealed after the whole-genome sequencing.

The sequencing library was prepared using the Nextera XT DNA sample preparation kit (Illumina, Inc., San Diego, CA, USA). Sequencing was done in paired-end mode with 2 × 300-bp reads via the MiSeq sequencer using the MiSeq reagent (600-cycle) kit version 3 (Illumina).

A total of 4,005,852 raw reads were generated and then assembled directly with no filtering according to quality using SPAdes version 3.11.1 (10) with k values of 21, 33, 55, 77, 99, and 127. The quality of the assembly was evaluated using QUAST version 5.0.0 (11), and genome annotation was accomplished using Prokka version 1.12 (12) and the RAST server (13).

Genome assembly yielded 185 contigs with a total length of 4,788,525 bp, an *N*_50_ value of 57,069 bp, and a G+C content of 66.14%. The average depth of coverage was 209.0×. The annotation of the contigs revealed that the strain possessed 4,262 genes encoding proteins and 76 RNA genes (70 tRNAs and 6 rRNAs). From a total of 4,262 identified proteins, 2,967 were classified as nonhypothetical proteins, while 1,929 sequences were assigned to SEED subsystems.

Using the JSpeciesWS online service (14), average nucleotide identity (ANI) values for the draft genome sequence of S. maltophilia MDMC339 compared to other *Stenotrophomonas* spp. were determined. The highest ANI value was 93.52% with S. maltophilia strain K279a (GenBank accession number NC_010943).

All software used during this study was run with default parameters.

The genome of Stenotrophomonas maltophilia MDMC339 contains genes that code for proteins induced by heat shock, notably *dnaK*, *dnaJ*, and *grpE*, and transcription repressor *hrcA* (15). In addition to heat shock proteins, the genome also contains genes involved in the response to cold shock (*cspA* and *cspD*) (16).

Genome analysis also reveals the presence of the *betA* and *betB* genes involved in the enzymatic conversion of choline and glycine betaine aldehyde to glycine betaine. The import of choline is done using high-affinity choline uptake gene *betT*, the sequence of which is present in four copies (17). Finally, the genome encodes proteins of the type VI secretion system, which is known for its role in interbacterial competition (18) and which could be useful in competition for environmental resources.

### Data availability.

This whole-genome shotgun project has been deposited at DDBJ/ENA/GenBank under the accession number RXLZ00000000. The version described in this paper is the first version, RXLZ01000000. The raw sequencing data are available from the Sequence Read Archive (SRA) under the accession number SRR11886254. The bacterial 16S rRNA marker gene sequenced during this study is available at the NCBI GenBank database under accession number KX013440.
